# [^18^F]PSMA-1007 PET/CT-based radiomics may help enhance the interpretation of bone focal uptakes in hormone-sensitive prostate cancer patients

**DOI:** 10.1007/s00259-025-07085-6

**Published:** 2025-01-28

**Authors:** Matteo Bauckneht, Giovanni Pasini, Tania Di Raimondo, Giorgio Russo, Stefano Raffa, Maria Isabella Donegani, Daniela Dubois, Leonardo Peñuela, Luca Sofia, Greta Celesti, Fabiano Bini, Franco Marinozzi, Francesco Lanfranchi, Riccardo Laudicella, Gianmario Sambuceti, Alessandro Stefano

**Affiliations:** 1 Department of Health Sciences (DISSAL), University of Genoa, Genoa, Italy; 2 Nuclear Medicine, IRCCS Ospedale Policlinico San Martino, Genoa, Italy; 3 Department of Mechanical and Aerospace Engineering (DIMA), Sapienza University of Rome, Rome, Italy; 4 Institute of Bioimaging and Complex Biological Systems - National Research Council (IBSBC - CNR), Cefalù, Italy; 5 Department of Experimental Medicine (DIMES), University of Genoa, Genoa, Italy; 6 Nuclear Medicine, Department of Biomedical and Dental Sciences and Morpho-Functional Imaging, University of Messina, Messina, Italy

**Keywords:** Prostate cancer, Prostate-specific membrane antigen, Positron Emission Tomography, Unspecific bone uptakes, Radiomics, Machine-learning

## Abstract

**Purpose:**

We hypothesised that applying radiomics to [^18^F]PSMA-1007 PET/CT images could help distinguish Unspecific Bone Uptakes (UBUs) from bone metastases in prostate cancer (PCa) patients. We compared the performance of radiomic features to human visual interpretation.

**Materials and methods:**

We retrospectively analysed 102 hormone-sensitive PCa patients who underwent [^18^F]PSMA-1007 PET/CT and exhibited at least one focal bone uptake with known clinical follow-up (reference standard). Using matRadiomics, we extracted features from PET and CT images of each bone uptake and identified the best predictor model for bone metastases using a machine-learning approach to generate a radiomic score. Blinded PET readers with low (*n* = 2) and high (*n* = 2) experience rated each bone uptake as either UBU or bone metastasis. The same readers performed a second read three months later, with access to the radiomic score.

**Results:**

Of the 178 [^18^F]PSMA-1007 bone uptakes, 74 (41.5%) were classified as PCa metastases by the reference standard. A radiomic model combining PET and CT features achieved an accuracy of 84.69%, though it did not surpass expert PET readers in either round. Less-experienced readers had significantly lower diagnostic accuracy at baseline (*p* < 0.05) but improved with the addition of radiomic scores (*p* < 0.05 compared to the first round).

**Conclusion:**

Radiomics might help to differentiate bone metastases from UBUs. While it did not exceed expert visual assessments, radiomics has the potential to enhance the diagnostic accuracy of less-experienced readers in evaluating [^18^F]PSMA-1007 PET/CT bone uptakes.

**Supplementary Information:**

The online version contains supplementary material available at 10.1007/s00259-025-07085-6.

## Introduction

Unspecific Bone Uptakes (UBUs) are incidental findings frequently observed in prostate cancer (PCa) patients undergoing Prostate Specific Membrane Antigen Positron Emission Tomography/Computed Tomography (PSMA PET/CT), particularly with [^18^F]PSMA-1007 [1–6]. UBUs represent a significant clinical challenge, as they can mimic metastatic bone lesions, making accurate interpretation difficult—especially for nuclear medicine physicians with less experience in PSMA PET reporting [7]. This creates a dilemma: overinterpretation may lead to stage migration, potentially resulting in unnecessary interventions or inappropriate treatment changes, depriving patients of curative options. Conversely, under-interpretation could result in overly aggressive treatments that are not curative [8, 9].

Recent advancements in machine-learning techniques and the rapid increase in computational power have given rise to radiomics [10]. Radiomics is a high-throughput approach that provides quantitative descriptors of tumour phenotypes in shape, statistical, and textural dimensions via image analysis, thus enabling better clinical decision-making while addressing the limitations of visual assessment alone. Numerous studies have shown that radiomic-based machine-learning methods may identify findings that are invisible to the human eye [10–13].

Based on this, in the present study, we hypothesised that applying radiomics to [^18^F]PSMA PET/CT images could improve the differentiation between UBUs and metastatic bone lesions in PCa patients. Additionally, we evaluated the potential additive value of radiomics to visual interpretation by PET readers with varying experience levels.

## Materials and methods

### Study population

We retrospectively analyzed a real-world series of [^18^F]PSMA-1007 PET/CT scans from patients with histology-proven, hormone-sensitive PCa, conducted at IRCCS Ospedale Policlinico San Martino for staging or restaging purposes, from the 05.08.2021 to the 14.03.2023. Bone lesions were identified by two authors of the manuscript (M.B. and R.L. with 11 and 9 years of experience, respectively) who were not involved in the imaging review process. Skeletal/bone marrow findings were included if they visually exhibited focal uptake greater than the surrounding background, regardless of the presence of a morphological correlate on CT. Patients with synchronous malignancies, known bone disorders, active inflammatory processes, or castration-resistant PCa were excluded. Supplementary Fig. 1 presents the patient selection flowchart. The study adhered to the Declaration of Helsinki and was approved by the local ethics committee (code 5/2023, database identifier 12914). All patients signed a general informed consent for retrospective studies.

### Imaging procedures, analyses and data collection

[^18^F]PSMA-1007 PET/CT scans were acquired following current guidelines [14]. All studies were performed using state-of-the-art equipment, including the Biograph Hirez 16 (Siemens Healthineers), Biograph MCT Flow (Siemens Healthineers), and Omni Legend 32 (GE Healthcare). Regardless of the scanner, CT images were acquired with parameters of 120 kV, 30–400 mA, and a pitch of 0.984:1/39.37. Two nuclear medicine physicians (M.B. and R.L.) interpreted, in consensus, the images according to the E-PSMA standardized reporting guidelines [15] to assess the presence or absence of [^18^F]PSMA-1007 focal bone uptakes. If uptake was present, the number and regional site were recorded. The point of maximum tracer uptake (assessed by measuring the maximum Standardized Uptake Value, SUVmax) was selected as the center of a volume of interest, drawn by the threshold method (45% SUVmax as previously described [7]) to calculate SUVmean. The same volume of interest was used on CT images to assess mean and maximum Hounsfield units (HUmean and HUmax, respectively). Clinical and laboratory data at the time of the [^18^F]PSMA-1007 PET/CT scan were collected from electronic medical records.

### Standard reference definition

No patient enrolled in the study underwent a bone biopsy. Instead, two authors not involved in the imaging review process (T.D.R. and L.S.) defined a composite standard reference based on clinical, biochemical, and imaging data, as previously described [7]. Specifically, changes in size, spontaneous disappearance, or the appearance of a CT correlate during imaging follow-up without any treatment were considered indirect confirmation of the presence or absence of bone metastases. Similarly, a decrease in prostate-specific antigen (PSA) of at least 50% after metastasis-directed therapy at a bone uptake site was considered indirect confirmation of underlying PCa. No minimum follow-up period was required if the reference standard could be definitively established. Patients with lesions classified as uncertain after clinical, biochemical, and imaging follow-ups were excluded from the analysis (*n* = 42, Supplementary Fig. 1).

### Radiomic features extraction and analysis

Using the matRadiomics tool [16], which incorporates the IBSI-compliant Pyradiomics [17] feature extractor, 1781 radiomic features were extracted from PET and CT images of each bone uptake, either without (original images) or with preprocessing (Laplacian of Gaussian and wavelet decomposition). Images were resampled to isotropic voxel (2 × 2 × 2 mm^3^) using the *sitkBSpline* interpolator. The *Bin Width* was set to 0.25, the *sigma* was set in a range between 0.5 and 5 with 0.5 increments, and *coif1* was chosen as the wavelet type. Features were processed through a preliminary machine-learning pipeline (100 repetitions), incorporating an 80%/20% split for training/testing, Least Absolute Shrinkage and Selection Operator (LASSO) for feature selection, and fivefold cross-validation for model optimization and testing. In total, six classifiers were tested: Discriminant Analysis (DA), Support Vector Machines (SVM), K-Nearest Neighbour (KNN), Neural networks (NN), Random Forest (RF) and AdaBoost (Boost). The preliminary pipeline was designed to collect a set of selected features, ranked by selection frequency. This approach facilitates the identification of optimal hyperparameters and the selection of the best candidate models. These models were then used to construct the ensemble model based on the average performance metrics, ensuring a robust and well-calibrated outcome. Among all selected features, CT features with a selection frequency between 30 and 70% were correlated with those exhibiting a selection frequency greater than 70% using the Pearson Correlation Coefficient (PCC) to create an optimal CT feature dataset. To evaluate the impact of PET features on model performance, PET features with a selection frequency between 30 and 70% were incrementally added to the CT feature dataset, one at a time. The performance metrics are reported as averages along with the 95% Confidence Interval (CI), computed using 10,000 bootstrapping. Statistical tests comparing the performance of radiomics and readers were performed using the Kruskal–Wallis test with 10,000 bootstrap samples, followed by the Sidak correction for multiple comparisons.

### Comparison between radiomics accuracy and PET readers’ performance

PET readers with varying levels of experience in PSMA PET interpretation were recruited for the study. This included readers with low experience (fewer than 30 prior scan interpretations; *n* = 2: D.D. and L.P.) and high experience (more than 300 prior scan interpretations; *n* = 2: S.R. and M.I.D.) [18]. All readers were blinded to the clinical history of patients. They were provided with the slice numbers to locate the focal bone uptake to be evaluated precisely. Readers were required to classify each bone uptake as either malignant or benign (UBU), with no option to categorise it as uncertain. During the interpretation process, they were permitted to qualitatively assess PET and CT images and extract relevant parameters, such as SUV or HU. To ensure an unbiased evaluation, prior PSMA PET examinations, if available, were not provided. Readers were instructed to base their reports solely on the current exam relevant to the study. After three months, the same readers re-evaluated the same PSMA PET-positive bone uptakes, again blinded to the clinical history, but this time with access to a continuous score reflecting the radiomic results (second round of reporting). This score was calculated by applying the best radiomic model, identified based on the F-score, to each bone uptake, producing a probability of bone metastasis expressed as a percentage. The F-score is a metric that combines precision (positive predictive value, PPV) and sensitivity to measure a model’s accuracy. Specifically, it is the harmonic mean of precision and sensitivity, and it helps assess a model's ability to correctly identify true positive (TP) cases while minimising false positives (FP) and false negatives (FN). Accordingly, to the following equation:

$${F}_{score}=\frac{2\left(precision\times sensitivity\right)}{precision+sensitivity}=\frac{2TP}{2TP+FP+ FN}$$

**Fig. 1:**
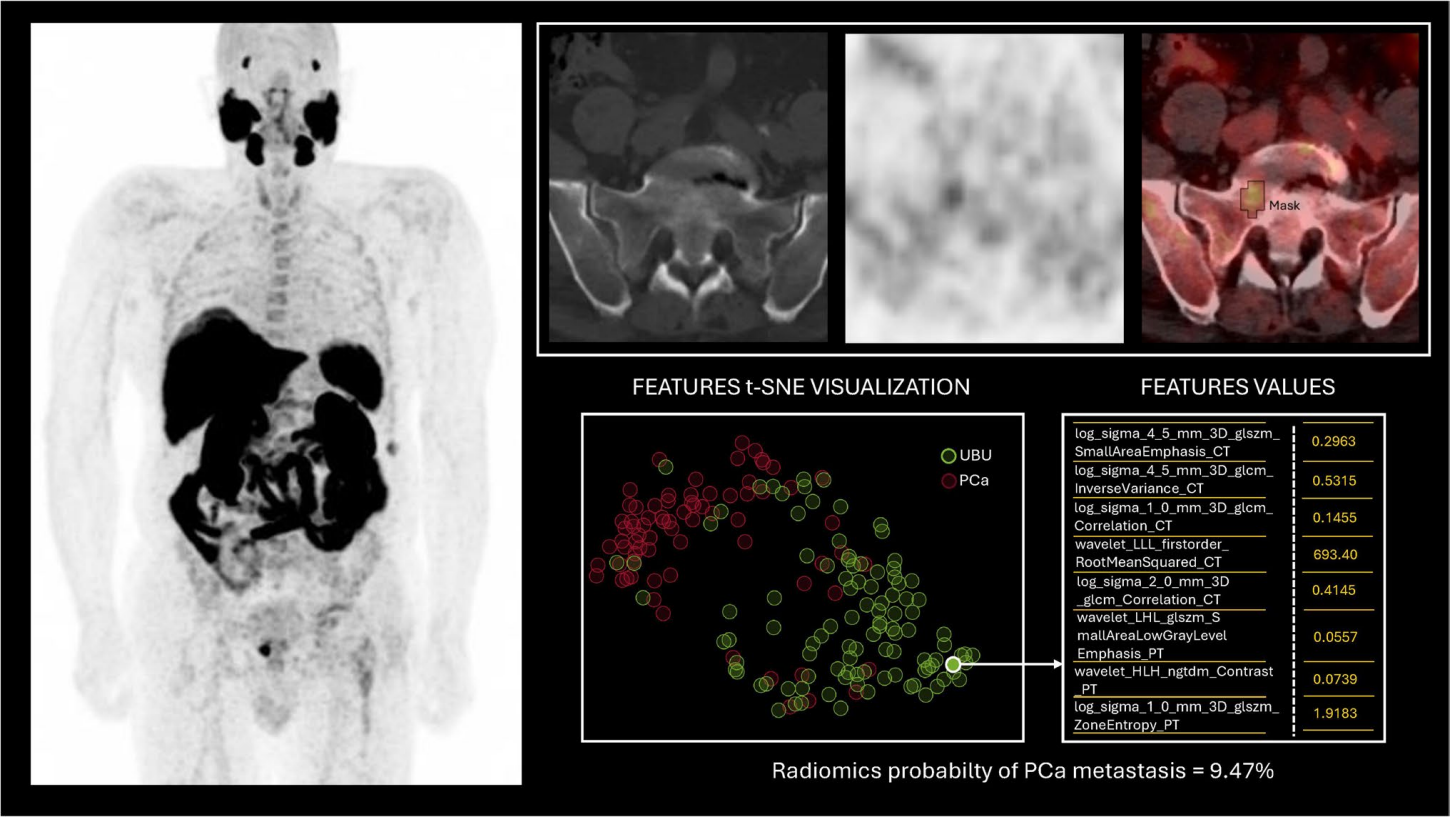
Radiomic Analysis of Focal [^18^F]PSMA-1007 Bone Uptake. *Legend:* The left panel shows the maximum intensity projection (MIP) of a [^18^F]PSMA-1007 PET/CT scan performed for primary staging in a high-risk PCa patient candidate for surgery. The right panel presents axial CT, PET, and hybrid PET/CT images (upper row), illustrating a focal bone uptake in the right sacrum with segmentation overlaid in black on the PET/CT images. In the lower section of the same panel, selected radiomic features are visualised using t-Distributed Stochastic Neighbor Embedding (t-SNE), with UBUs distribution in green and metastatic PCa lesions in red, alongside the associated feature values for the considered right sacral uptake. After the PET/CT scan, the patient underwent radical prostatectomy followed by long-term PSA suppression (PSA < 0.01 ng/mL twelve months after surgery). Based on clinical history, the sacral tracer uptake was interpreted as UBU in the reference standard. During the initial assessment, expert readers correctly identified the right sacral uptake as non-metastatic. In contrast, one of the two less-experienced PET readers classified it as suspicious for PCa metastasis. Indeed, the radiomic analysis revealed a low probability of bone metastasis, with a radiomic score of 9.47%. In the second round of revision, all readers correctly classified the sacral uptake as non-metastatic

During the second round of analysis, readers were provided with a continuous score, reflecting the weighted probability of each uptake being metastatic PCa [19]. This probability was calculated using the following equation:

$${w}_{probability}=\frac{\sum_{i=1}^{n}{w}_{i}\times {p}_{i}}{\sum_{i=1}^{n}{w}_{i}}$$

where *n* is the number of models constituting the ensemble machine-learning model, w_*i*_ is the Area Under the Curve (AUC) of the i-th model, and p_*i*_ is the posterior probability that the i-th model is assigned to that observation. This ensemble approach allows for a robust combination of predictions from multiple models to enhance classification accuracy. Figure 1 has been included to provide a visual representation of the radiomic analysis, highlighting the extraction of radiomic features and the calculation of the radiomic score for a focal bone uptake.

## Results

### Patient’s and bone focal uptakes characteristics

Table 1 summarises the clinical characteristics of the 102 enrolled patients, while Table 2 details the imaging features of the 178 bone [^18^F]PSMA-1007 focal uptakes analysed in this study. Of the 178 focal uptakes, 77 (43.2%) demonstrated a morphological correlate on CT, with 53/77 (68.8%) visually classified as suspicious for PCa bone metastases. Finally, according to the reference standard, 74 (41.5%) focal bone uptakes (with/without morphological alterations) were ultimately considered PCa metastases.

**Table 1:** Clinical characteristics of the enrolled patients

Clinical setting	
Primary staging	88 (86.3%)
Restaging	14 (13.7%)
ISUP grade group	
1	5 (4.9%)
2	29 (28.5%)
3	26 (25.5%)
4	22 (21.6%)
5	11 (10.7%)
Missing	9 (8.8%)
PSA at PET/CT (ng/mL), mean ± SD	28.9 ± 115.8
ADT at PET/CT, *n* (%)	
No	90 (88.3%)
Yes	12 (11.7%)
Other systemic treatments at PET/CT, *n* (%)	
No	102 (100%)
Yes	0 (0%)
[^18^F]PSMA-1007 injected dose (MBq), mean ± SD	309.1 ± 48.1
Uptake time (min), mean ± SD	111.1 ± 22.4

*ADT: Androgen Deprivation Therapy; ISUP: International Society of Urological Pathology; PET/CT: Positron Emission Tomography/Computed Tomography; PSA: Prostate-Specific Antigen; PSMA: Prostate-Specific Membrane Antigen; SD: Standard Deviation*

**Table 2:** Imaging data for each bone [^18^F]PSMA-1007 uptake

	Bone focal uptake	UBUs (*n* = 104)	Bone metastases (*n* = 74)	*p*-value
Bone uptake site				
Ribs	48 (27%)	32 (18%)	16 (9%)	
Sternum	2 (1.1%)	1 (0.6%)	1 (0.6%)	
Spine	58 (32.6%)	36 (20.2%)	22 (12.3%)	
Pelvis	53 (29.8%)	29 (16.3%)	24 (13.5%)	ns
Homer	3 (1.7%)	2 (1.1%)	1 (0.6%)	
Femur	8 (4.5%)	2 (1.1%)	6 (3.4%)	
Skull	2 (1.1%)	0 (0%)	2 (1.1%)	
Other sites	4 (2.2%)	2 (1.1%)	2 (1.1%)	
CT correlate at visual inspection	77 (43.3%)	26 (14.6%)	51 (28.6%)	< 0.0001
HU_mean_, mean ± SD	234.9 ± 127.3	209.7 ± 108.8	270.2 ± 142.7	0.0016
HU_max_, mean ± SD	696.1 ± 277.7	629.7 ± 269.1	789.4 ± 264	0.0001
SUV_max_, mean ± SD	9 ± 8.5	5 ± 2	14.6 ± 10.8	< 0.0001

*CT: Computed tomography; HU: Hounsfield Units; ns: Non-significant; SD: Standard Deviation; SUV: Standardized Uptake Value; UBUs Unspecific bone uptakes*

### Radiomic features predicting bone metastases

**Fig. 2:**
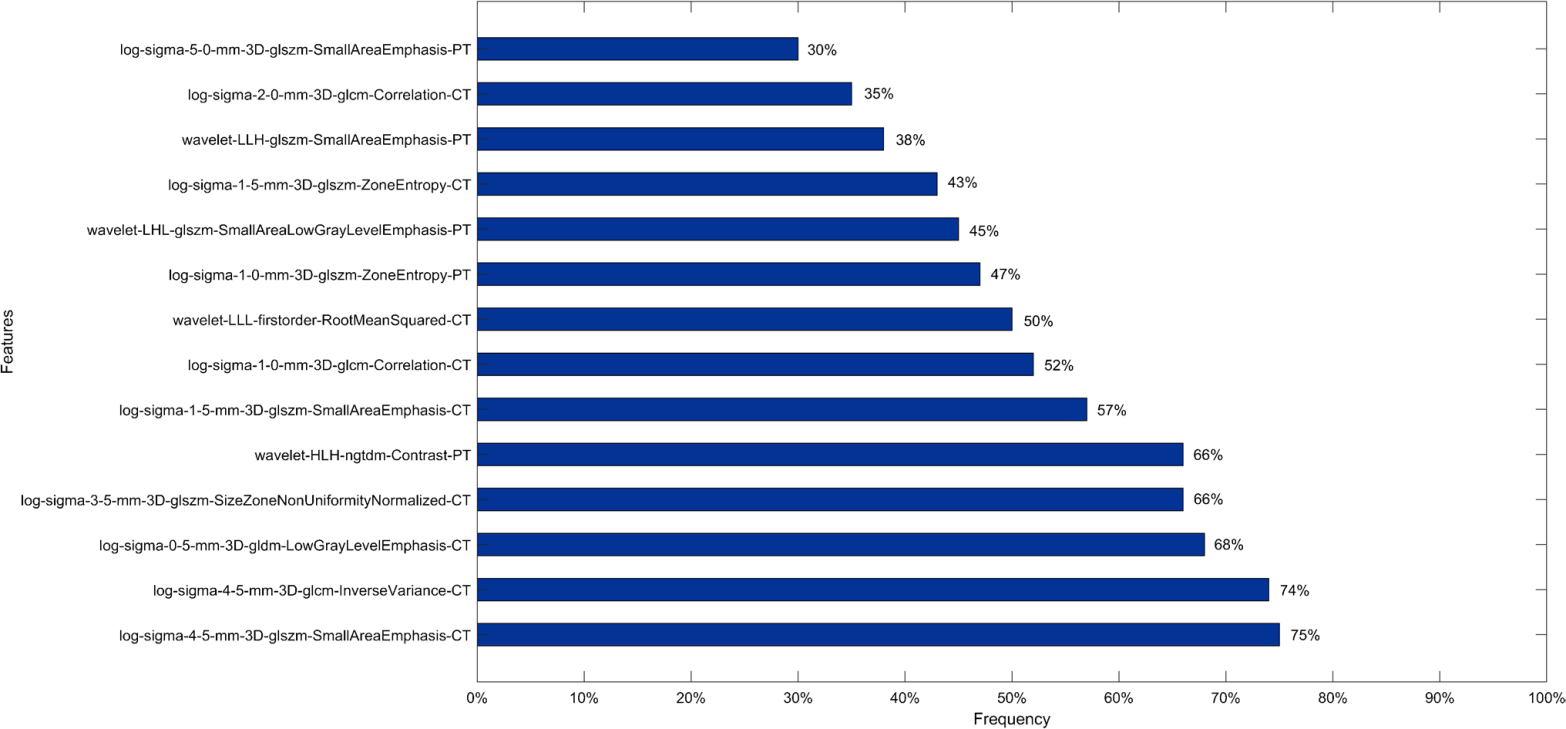
The fourteen radiomic features selected based on the frequency of occurrence ranging between 30 and 70%

The preliminary analysis revealed that fourteen features (9 from CT, and 5 from PET) had a selection frequency greater than 30%, as shown in Fig. 2. However, only two CT features, 'log_sigma_4_5_mm_3D_glszm_SmallAreaEmphasis' and 'log_sigma_4_5_mm_3D_glcm_InverseVariance_CT,' exhibited a selection frequency greater than 70%. Among the CT features with selection frequencies between 30 and 70%, only three CT-based features had a PCC below 0.3, namely a linear correlation (Table 3). Consequently, the final CT feature dataset consisted of just five features. The highest selection frequency for PET-based features was 66%, which was reached by the 'wavelet_HLH_ngtdm_Contrast' feature.

**Table 3:** The seven Computed Tomography (CT) features selected based on their selection frequency ranging between 30 and 70%

Feature	Frequency	PCC
log_sigma_0_5_mm_3D_gldm_LowGrayLevelEmphasis_CT	68%	0.69
log_sigma_3_5_mm_3D_glszm_SizeZoneNonUniformityNormalized_CT	66%	0.74
log_sigma_1_5_mm_3D_glszm_SmallAreaEmphasis_CT	57%	0.69
log_sigma_1_0_mm_3D_glcm_Correlation_CT	52%	**0.24**
wavelet_LLL_firstorder_RootMeanSquared_CT	50%	**0.17**
log_sigma_1_5_mm_3D_glszm_ZoneEntropy_CT	43%	0.75
log_sigma_2_0_mm_3D_glcm_Correlation_CT	35%	**0.29**

The Pearson Correlation Coefficient (PCC) was calculated to assess the relationship between these features. Features with a PCC ≤ 0.3 are highlighted in bold, as this indicates a low correlation, suggesting that these three features provide unique, non-redundant information to the model

The ensemble model included three distinct DA classifiers, one SVM classifier, and three NN classifiers. From the pool of hyperparameters, the most frequently selected (median values) were chosen, and the detailed parameters are listed in the Supplementary Materials (Supplementary Table 1 for DA classifiers, Supplementary Table 2 for SVM, and Supplementary Table 3 for NN classifiers). The model was then trained on various datasets composed of selected features, including a set containing only CT features (CT model) and others combining CT and PET features (CT + PET models). The CT + PET1 model incorporated CT features and the highest-ranked PET feature based on selection frequency. In contrast, the CT + PET2 model included CT features, the top two most frequently selected PET features, and so on. As a result, six distinct models were generated, each representing a different combination of CT and PET feature sets, designed to explore their predictive potential (CT model, CT + PET1, CT + PET2, CT + PET3, CT + PET4, and CT + PET5).

**Fig. 3:**
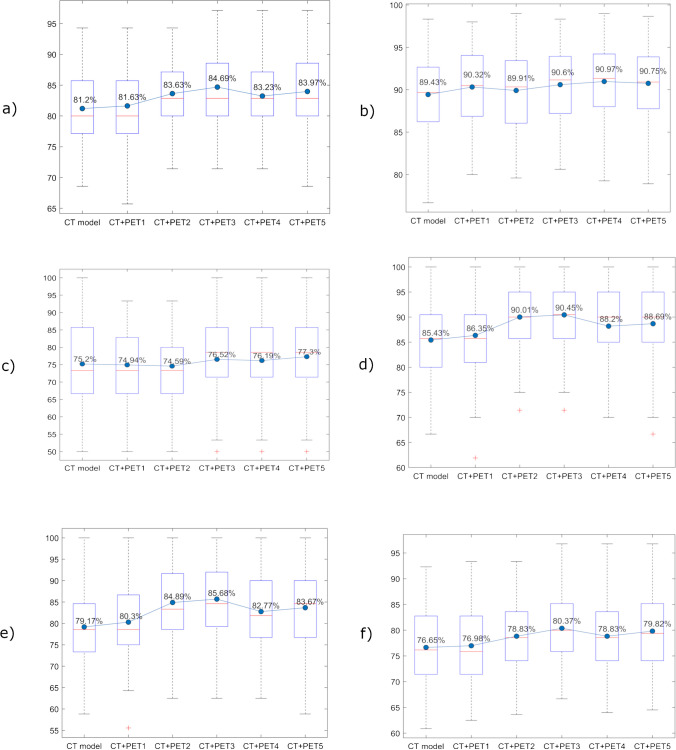
Boxplots for each model and for each metric. *Legend:* The panels display **a**) accuracy, **b**) Area Under the Curve (AUC), **c**) sensitivity, **d**) specificity, **e**) precision, **f**) F-score. The blue filled circle represents the average value, while the red line the median value. These metrics are computed on test sets. *CT: Computed Tomography; PET: Positron Emission Tomography*

On the test sets, the CT + PET3 model achieved the highest average accuracy (84.69%), specificity (90.45%), precision (85.68%), and F-score (80.37%). The CT + PET4 model reached the highest average AUC (90.97%), while the CT + PET5 model obtained the highest average sensitivity (77.30%). Boxplots comparing model performance for each metric are shown in Fig. 3, and the full performance metrics, along with 95% CI, are provided in Table 4.

**Table 4:** Average performance of each of the 6 models for each metric, together with the 95% CI

Model	Accuracy	AUC	Sensitivity	Specificity	Precision	F-score
CT	81.20% (80.09 −82.34)	89.43% (88.50–90.30)	75.20% (73.20–75.20)	85.43% (84–86.80)	79.17% (77.44–80.92)	76.65% (75.18–78.08)
CT + PET1	81.63% (80.57–82.71)	90.32% (89.50–91.2)	74.94% (72.90–76.80)	86.35% (84.90–87.70)	80.30% (78.63–81.98)	76.98% (75.62–78.39)
CT + PET2	83.63% (82.60–84.69)	89.91% (89–90.80)	74.59% (72.60–76.50)	90.01% (88.70–91.20)	84.89% (83.18–86.54)	78.83% (77.45–80.24)
CT + PET3	84.69% (83.66–85.71)	90.60% (89.70–91.40)	76.52% (74.60–78.30)	90.45% (89.20–91.60)	85.68% (84.03–87.36)	80.37% (79.03–81.74)
CT + PET4	83.23% (82.17–84.40)	90.97% (90.20–91.80)	76.29% (74.20–78.20)	88.20% (86.90–89.50)	82.77% (81.09–84.48)	78.83% (77.44–80.32)
CT + PET5	83.97% (82.86–85.09)	90.75% (89.90–91.60)	77.30% (75.30–79.30)	88.69% (87.30–90)	83.67% (81.95–85.49)	79.82% (78.39–81.25)

*AUC: Area Under the Curve; CI: Confidence Interval; CT: Computed Tomography; PET: Positron Emission Tomography*

These metrics are averaged over test sets

### Comparison between radiomic accuracy and PET readers' performance

We compared the performance of the best radiomic model reaching the higher F-score (CT + PET3) with the readers' visual assessments. Radiomics did not outperform expert PET readers, achieving a diagnostic accuracy of 92.1%, sensitivity of 89.19%, specificity of 94.23%, precision of 91.67%, and an F-score of 90.41%. However, the diagnostic accuracy of less-experienced readers was significantly lower compared to radiomics: 83.7% for one reader (*p* < 0.05), and 54.4% for the other (*p* < 0.01). Precision was also lower for the less-experienced readers, resulting in 77.78% and 47.62%, respectively (both *p* < 0.05). While one less-experienced reader achieved a higher F-score than the radiomics model (81.29%, *p* < 0.05), the other reader’s F-score was significantly lower at 63.35% (*p* < 0.05).

### Addition of the radiomic model to visual assessment according to PET readers' performance

**Fig. 4:**
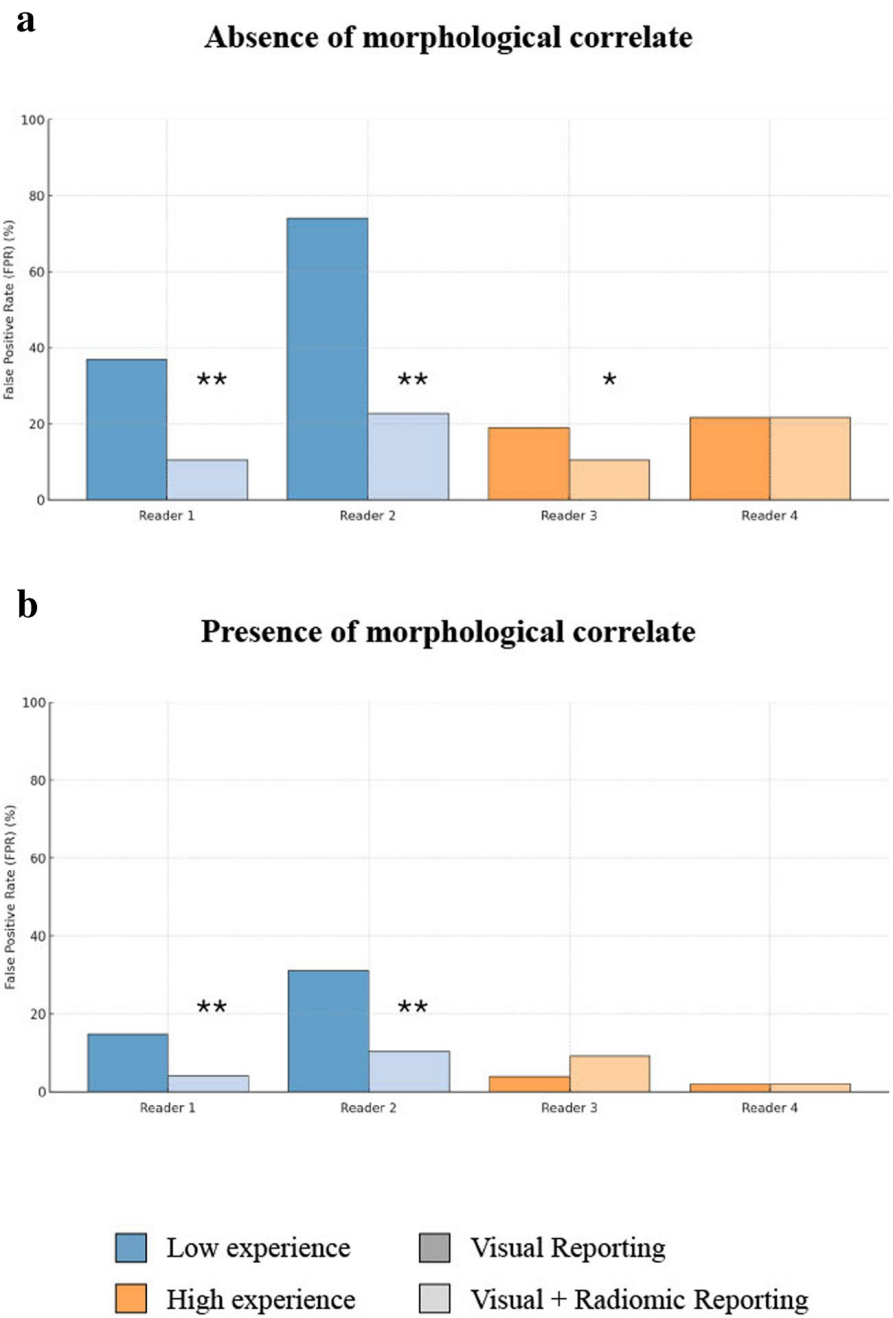
Impact of Morphological Correlates and Radiomic Support on False Positive Rates Across Reader Experience Levels. *Legend:* The charts illustrate the False Positive Rate (FPR) in different reporting conditions for readers with varying experience levels. Panel a depicts the FPR in the absence of morphological correlates, while Panel b highlights the FPR when morphological correlates are present. Without morphological correlates on CT, radiomic support significantly reduces the FPR, particularly for less experienced readers (Panel **a**). When morphological correlates are present, the FPR decreases across all reporting conditions, with less pronounced differences between visual-only and radiomic-augmented assessments. Nevertheless, radiomics continues to provide added value for less experienced readers in this context (Panel **b**). * = *p* < 0.05 vs. visual reporting; ** = *p* < 0.01 vs. visual reporting

The addition of radiomics to the visual assessment of bone uptakes significantly improved diagnostic accuracy, specificity (or true negative rate), precision, and the F-score for less-experienced readers in the second round of reporting. Sensitivity significantly increased for Reader 2 but not for Reader 1. Differently, the addition of radiomics did not significantly impact the performance of expert readers. The readers' performances across both reporting rounds are summarised in Table 5. Notably, the presence of a morphological correlate on CT influenced the added value of radiomics in visual assessment (Fig. 4, Supplementary Table 4). In the absence of morphological correlates, low-experience readers exhibited significantly higher false positive rates (FPR, defined as the ratio of false positives to all positives) than high-experience readers. In this scenario, radiomics support substantially reduced the FPR, with the greatest improvements observed in low-experience readers. High-experienced readers also benefited from radiomics augmentation, though the effect was less pronounced. Conversely, morphological correlates lowered the FPR across all readers and reporting conditions. While radiomics support continued to enhance performance for low-experience readers, its impact became negligible for experts.

**Table 5:** Readers' performances expressed in accuracy, sensitivity, specificity, precision and F-score

	Accuracy	Sensitivity	Specificity	Precision	F-score
Visual reporting					
Reader 1—Low experience (< 30)	83.71%	85.14%	82.69%	77.78%	81.29%
Reader 2—Low experience (< 30)	54.49%	94.59%	25.96%	47.62%	63.35%
Reader 3—High experience (> 300)	92.13%	89.19%	94.23%	91.67%	90.41%
Reader 4—High experience (> 300)	92.13%	89.19%	94.23%	91.67%	90.41%
Visual + radiomic reporting					
Reader 1—Low experience (< 30)	91.01%*	85.14%	95.19%**	92.65%**	88.73%*
Reader 2—Low experience (< 30)	84.27%**	78.38%**	88.46%**	82.86%**	80.56%**
Reader 3—High experience (> 300)	90.45%	87.84%	92.31%	89.04%	88.44%
Reader 4—High experience (> 300)	89.33%	85.14%	92.31%	88.73%	86.90%

* = *p* < 0.05 vs. visual reporting; ** = *p* < *0.01 vs. visual reporting*

## Discussion

In clinical practice, misinterpreting UBUs as metastatic lesions (and viceversa) can lead to disease over/down-staging, which may result in missed opportunities for curative treatments or unnecessary escalation of therapy [20–23]. This highlights the importance of accurate tools for distinguishing malignant and benign bone lesions in PCa patients undergoing [^18^F]PSMA-1007 PET/CT. To our knowledge, this is the first study to apply radiomics in this clinical context.

A strength of this study is comparing radiomic assessment of bone focal uptake with human evaluation by PET readers with varying levels of experience. Overall, our findings underscore the critical role of radiomic support in improving diagnostic accuracy, particularly for less experienced readers and in challenging scenarios where morphological correlates are absent. The superior performance of expert PET readers is not surprising and aligns with previous studies. Seifert et al. [5] retrospectively analysed a real-world cohort of 792 patients imaged for restaging purposes with either [^68^ Ga]Ga-PSMA-11 (*n* = 383) or [^18^F]PSMA-1007 (*n* = 409) at a single high-volume PET/CT centre. They found no difference in the detection rate of bone metastases between the two cohorts, suggesting that experienced nuclear medicine physicians can identify the UBU pattern and recognise lesions as unspecific. In a study by our group [7], we developed a composite score that included clinical and imaging characteristics to improve the reporting of focal bone uptake. This score increased specificity for less experienced PET readers without significantly improving the reporting performance of their more experienced counterparts. Although the exclusive impact on less experienced readers may seemingly diminish the overall significance of our study, it highlights a critical clinical issue. Less experienced readers are more prone to erroneous interpretations, which can lead to the overstaging of PCa patients and, ultimately, inappropriate changes in treatment plans with potential negative effects on oncological outcomes [24]. The application of radiomics could help mitigate this issue by providing objective, data-driven support and ensuring patients receive the most appropriate care.

Our analysis demonstrated that CT features were more stable than PET features, exhibiting higher selection frequencies. Notably, two CT-based features reached the highest selection frequencies, at 75% and 74%. However, many CT features contained redundant information, with only three features showing no correlation with the two most frequently selected ones. Consequently, the final CT feature set included five features: 'log_sigma_4_5_mm_3D_glszm_SmallAreaEmphasis,' 'log_sigma_4_5_mm_3D_glcm_InverseVariance,' 'log_sigma_1_0_mm_3D_glcm_Correlation' (PCC = 0.24), 'wavelet_LLL_firstorder_RootMeanSquared' (PCC = 0.17), and 'log_sigma_2_0_mm_3D_glcm_Correlation' (PCC = 0.29). Despite this, models trained using only CT features were less accurate than those combining both PET and CT features, suggesting that adding PET data improved the discrimination of bone metastases. Adding PET features, based on their selection frequency, enhanced the ensemble model’s performance. Accuracy increased from 81.20% to 84.69%, AUC improved from 89.43% to 90.97%, precision (PPV) rose from 79.17% to 85.68%, and the F-score (true positive rate) increased from 76.65% to 80.37%. The observed predictive capability of CT features is particularly noteworthy given the improvement of the presented radiomic model over visual assessment alone in interpreting bone focal uptakes without a discernible CT correlate during visual inspection. By extracting and analysing quantitative features from CT images, radiomics can potentially identify subtle structural or textural alterations imperceptible to the human eye. These features may reflect underlying pathophysiological changes that precede or remain undetectable over time. This finding underscores the potential of radiomics as a valuable complement to visual assessment, particularly in diagnostically challenging cases.

The best model, based on the F-score, incorporated three PET features in addition to the CT features (the CT + PET3 model): 'wavelet_HLH_ngtdm_Contrast,' 'log_sigma_1_0_mm_3D_glszm_ZoneEntropy,' and 'wavelet_LHL_glszm_SmallAreaLowGrayLevelEmphasis.' The F-score was chosen as the primary metric because it provides a balanced measure of sensitivity and precision. Additionally, the CT + PET3 model achieved the highest accuracy at 84.69%. The pipeline used in this study was designed to ensure robustness and clarity in radiomic feature selection, with the ensemble model approach yielding more reliable results than individual models. Interestingly, all three selected PET features describe the heterogeneity of tracer uptake within the bone. Small Area Low Gray Level Emphasis (SALGLE) quantifies the proportion of smaller-sized zones with lower grey-level values in the image. Zone Entropy (ZE) reflects the uncertainty or randomness in the distribution of zone sizes and grey levels, with higher values indicating heterogeneity in texture patterns. Contrast measures spatial intensity changes, which are more pronounced in images with a wide range of grey levels and significant differences between adjacent voxels. This suggests that PET features may better capture the complexity of bone lesions, leading to more accurate differentiation between malignant and benign findings.

This study has some limitations. First, the absence of an external test set prevented us from validating our findings. However, the single-center nature of the study ensured standardized and reproducible PET/CT acquisition protocols, minimizing the technical impact on the reproducibility of radiomic features. The reference standard used in this study was not histopathological but relied on clinical, biochemical, and imaging data. While patients with uncertain bone lesions at follow-up were excluded, this remains a limitation that should be considered when interpreting the results. Additionally, the study's sample size was limited to 102 patients. In future work, we plan to continue collecting more patient data to further train and validate our model's performance in a larger cohort, including external confirmation. Moreover, this study did not consider additional forms of data harmonisation beyond isotropic resampling. These could be explored in future work, particularly to evaluate scanners' impact on radiomics analysis. We intentionally decided not to harmonise feature data using methods such as ComBat, as these methods are typically applied before train-test splitting. This decision was made because accessing the learned parameters without modifying the source code is not straightforward, and such parameters must be estimated on the training set and subsequently applied to the test set. Our primary objective was to develop a robust machine-learning model while strictly preventing data leakage. A further methodological consideration regards the sole inclusion criterion: the presence of at least one bone focal uptake with intensity greater than the surrounding bone marrow, regardless of a corresponding CT correlate. This may have contributed to the higher proportion of metastases observed in our cohort compared to other studies [6]. However, this approach reflects real-world clinical scenarios where both overt metastases with morphological correlates and more subtle lesions are encountered. Similarly, focal bone uptakes were visually identified by two expert readers without applying quantitative uptake intensity thresholds. This strategy aimed to develop a radiomic method suited to clinical practice, avoiding additional selection biases. Finally, we acknowledge potential heterogeneity in our cohort due to ongoing therapy, which may influence PSMA expression [25]. However, this bias is mitigated by focusing on patients with hormone-sensitive PCa, with only a small percentage receiving ADT at the time of imaging.

## Conclusion

[^18^F]PSMA-1007 PET/CT-based radiomics models can distinguish bone metastases from UBUs with high accuracy. Although radiomics did not outperform the visual assessments of expert PET readers in interpreting bone [^18^F]PSMA-1007 focal uptakes, it has the potential to enhance the interpretive accuracy of less-experienced readers.

## Supplementary Information

Below is the link to the electronic supplementary material.

Supplementary file1 (DOCX 18 KB)

Supplementary file2 (PPTX 32 KB)

## Data Availability

M.B. is responsible for data integrity and accuracy of the analyses. Raw data are available upon specific requests.
