# Supplementary material for: [18F]PSMA-1007 PET/CT-based radiomics may help enhance the interpretation of bone focal uptakes in hormone-sensitive prostate cancer patients
Source: Eur J Nucl Med Mol Imaging. 2025 Jan 28;52(6):2076–86. doi: 10.1007/s00259-025-07085-6 (PMC12014812; doi:10.1007/s00259-025-07085-6)
Supplement: Supplementary file 1 — Supplementary file1 (DOCX 18 KB) [file 259_2025_7085_MOESM1_ESM.docx]

**Supplementary Materials**

Supplementary Tables 1-3 are related to the chosen hyperparameters for the different models used in the final ensemble, as described in the manuscript.

Supplementary Table 1: The chosen hyperparameters for the DA classifiers

| Kernel function | gamma | delta |
| --- | --- | --- |
| linear | 0.5147 | 0.0056 |
| diaglinear | 0.5133 | 0.0114 |
| diagquadratic | 0 | 0 |

Supplementary Table 2: The chosen hyperparameters for the SVM classifier

| Kernel function | Box constraint | Kernel scale |
| --- | --- | --- |
| gaussian | 1.0516 | 73.2936 |

Supplementary Table 3: The chosen hyperparameters for the NN classifiers

| Activation | Fully connected layers | Weight initializer | Bias Initializer | Lambda |
| --- | --- | --- | --- | --- |
| sigmoid | 1 | he | zeros | 0.0118 |
| relu | 1 | he | ones | 0.0133 |
| tanh | 1 | he | ones | 0.0220 |

**Supplementary Table 4:** False Positive Rate (FPR) and True Positive Rate (TPR) for visual-only reporting and visual + radiomics reporting in the absence or presence of a morphological correlate

|  | **Absence of morphological correlate** | | **Presence of morphological correlate** | |
| --- | --- | --- | --- | --- |
|  | **FPR** | **TPR** | **FPR** | **TPR** |
| **Visual Reporting** |  |  |  |  |
| Reader 1 - Low experience (<30) | 37% | 63% | 14.8% | 85.2% |
| Reader 2 - Low experience (<30) | 74% | 26% | 31.1% | 68.9% |
| Reader 3 - High experience (>300) | 19% | 81% | 3.9% | 96.1% |
| Reader 4 - High experience (>300) | 21.7% | 78.3% | 2% | 98% |
| **Visual +** **radiomics reporting** |  |  |  |  |
| Reader 1 - Low experience (<30) | 10.5% | 89.5% | 4.1% | 95.9% |
| Reader 2 - Low experience (<30) | 22.7% | 77.3% | 10.4% | 89.6% |
| Reader 3 - High experience (>300) | 10.5% | 89.5% | 9.3% | 90.7% |
| Reader 4 - High experience (>300) | 21.7% | 78.3% | 2.1% | 97.9% |
