# Supplementary material for: [18F]PSMA-1007 PET/CT-based radiomics may help enhance the interpretation of bone focal uptakes in hormone-sensitive prostate cancer patients
Source: Eur J Nucl Med Mol Imaging. 2025 Jan 28;52(6):2076–86. doi: 10.1007/s00259-025-07085-6 (PMC12014812; doi:10.1007/s00259-025-07085-6)
Supplement: Supplementary file 2 — Supplementary file2 (PPTX 32 KB) [file 259_2025_7085_MOESM2_ESM.pptx]

## Slide 1
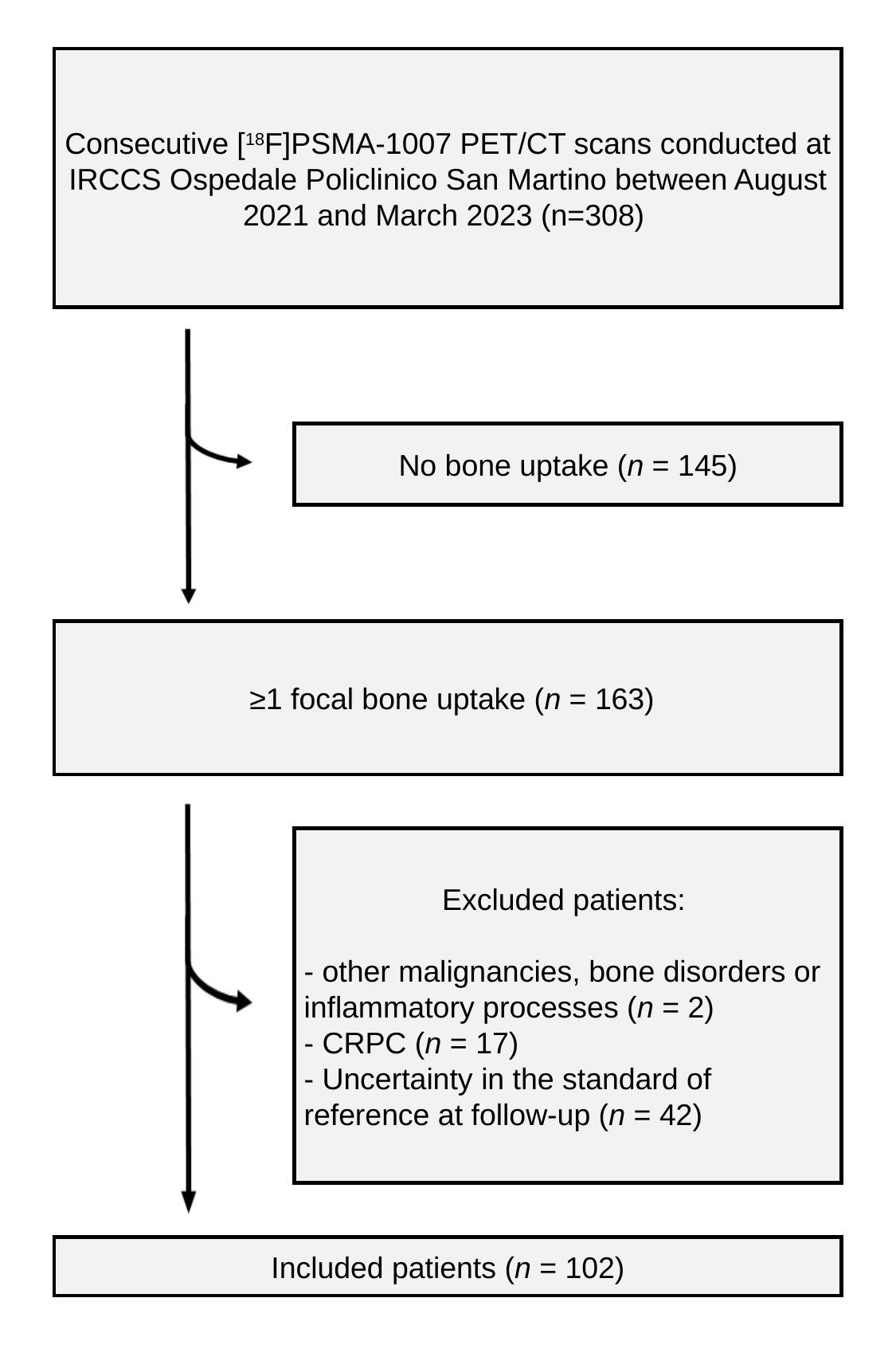

Consecutive [18F]PSMA-1007 PET/CT scans conducted at IRCCS Ospedale Policlinico San Martino between August 2021 and March 2023 (n=308)
No bone uptake (n = 145)
 ≥1 focal bone uptake (n = 163)
Excluded patients:
- other malignancies, bone disorders or inflammatory processes (n = 2)
- CRPC (n = 17)
- Uncertainty in the standard of reference at follow-up (n = 42)
Included patients (n = 102)
